# Supplementary material for: Depletion of Nsd2-mediated histone H3K36 methylation impairs adipose tissue development and function
Source: Nat Commun. 2018 May 4;9:1796. doi: 10.1038/s41467-018-04127-6 (PMC5935725; doi:10.1038/s41467-018-04127-6)
Supplement: Supplementary file 1 — Supplementary Information [file 41467_2018_4127_MOESM1_ESM.pdf]

Title: Depletion of Nsd2-mediated histone H3K36 methylation impairs adipose tissue development and function

Zhuang et al.

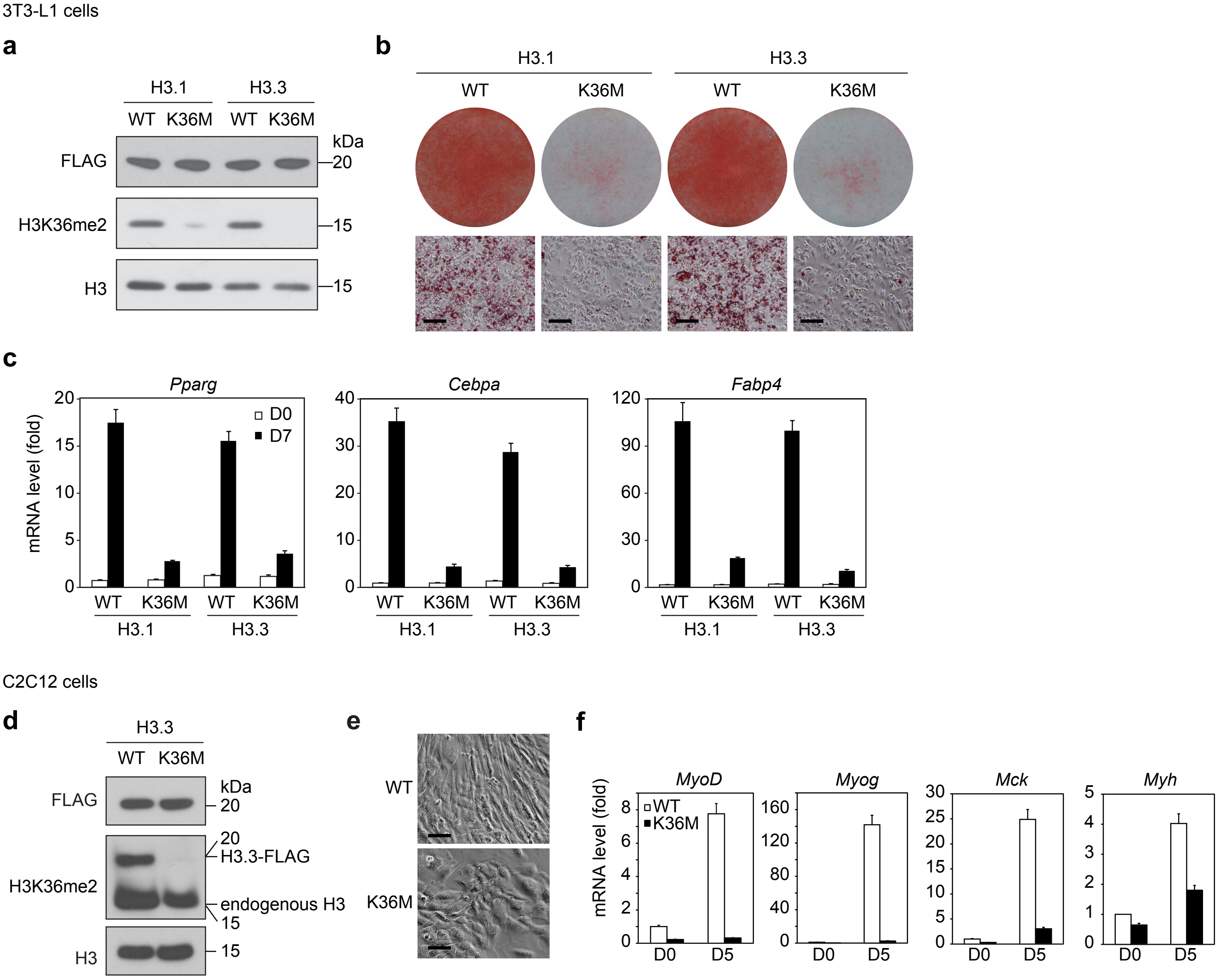

Supplementary Figure 1. H3.3K36M inhibits adipogenesis and myogenesis. (**a-c**) 3T3-L1 cells were infected with retroviral vector pQCXIP expressing FLAG-tagged WT or K36M of histone H3.1 or H3.3, followed by adipogenesis assay. (**a**) Western blot of FLAG-tagged histone H3 and H3K36me2 before adipogenesis. (**b**) Oil Red O staining at D7 of adipogenesis. Scale bars = 30 µm. (**c**) qRT-PCR of *Pparg*, *Cebpa* and *Fabp4* expression at D0 and D7 of adipogenesis. (**d-f**) C2C12 cells were infected with retroviral vector pQCXIP expressing FLAG-tagged WT or K36M of histone H3.3, followed by myogenesis assay. (**d**) Western blot of FLAG-tagged histone H3.3 and H3K36me2 before myogenesis. (**e**) Cell morphologies were observed under a microscope at day 5 (D5) of myogenesis. Scale bars = 20 µm. (**f**) qRT-PCR analysis of myogenic gene expression at day 0 (D0) and D5 of myogenesis. qRT-PCR data are presented as means ± SEM. Three technical replicates from a single experiment were used.

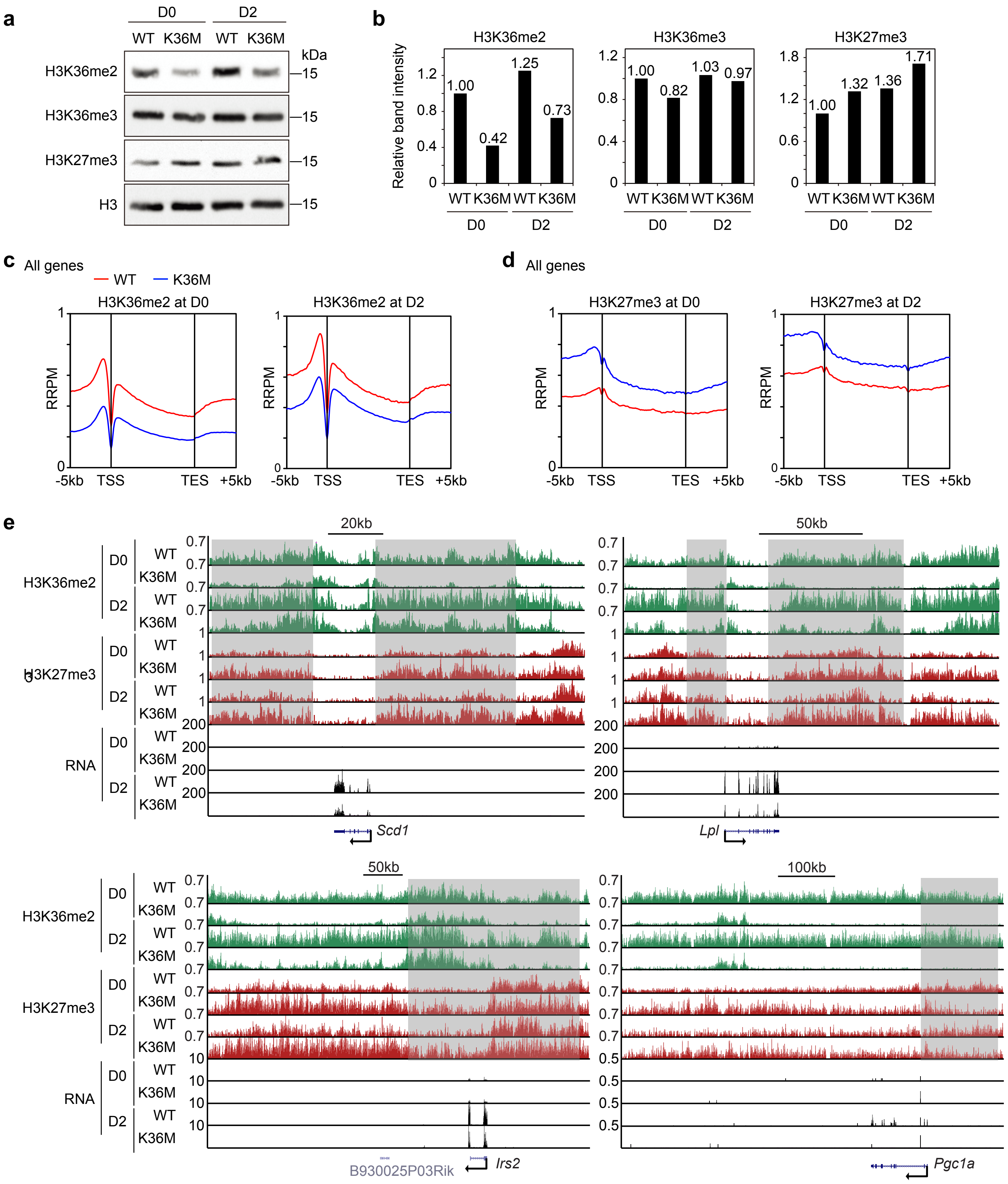

Supplementary Figure 2. Depleting H3K36 methylation by H3.3K36M increases H3K27me3 to prevent the induction of adipogenic genes. Immortalized brown preadipocytes were infected with retroviral vector pQCXIP expressing FLAG-tagged WT or K36M mutant histone H3.3, followed by adipogenesis assay. Cells were collected at indicated time points for Western blot,

ChIP-Seq of H3K36me2 and H3K27me3, and RNA-Seq analyses. (**a**) Western blot of histone methylations at D0 and D2 of adipogenesis. (**b**) Quantification of Western blot data shown in (**a**). Band intensities were quantified using ImageLab software and normalized to histone H3 levels. Relative histone methylation levels were compared between WT and K36M expressing cells. (**c-d**) Average profiles of H3K36me2 (**c**) and H3K27me3 (**d**) on gene body of all genes in WT and K36M expressing cells at D0 (left panel) and D2 (right panel) of adipogenesis. TSS, transcription start site; TES, transcription end site; RRPM, reference-adjusted reads per million. (**e**) Genome browser views of ChIP-Seq and RNA-Seq data on *Scd1*, *Lpl*, *Irs2* and *Pgc1a* loci during adipogenesis are shown.

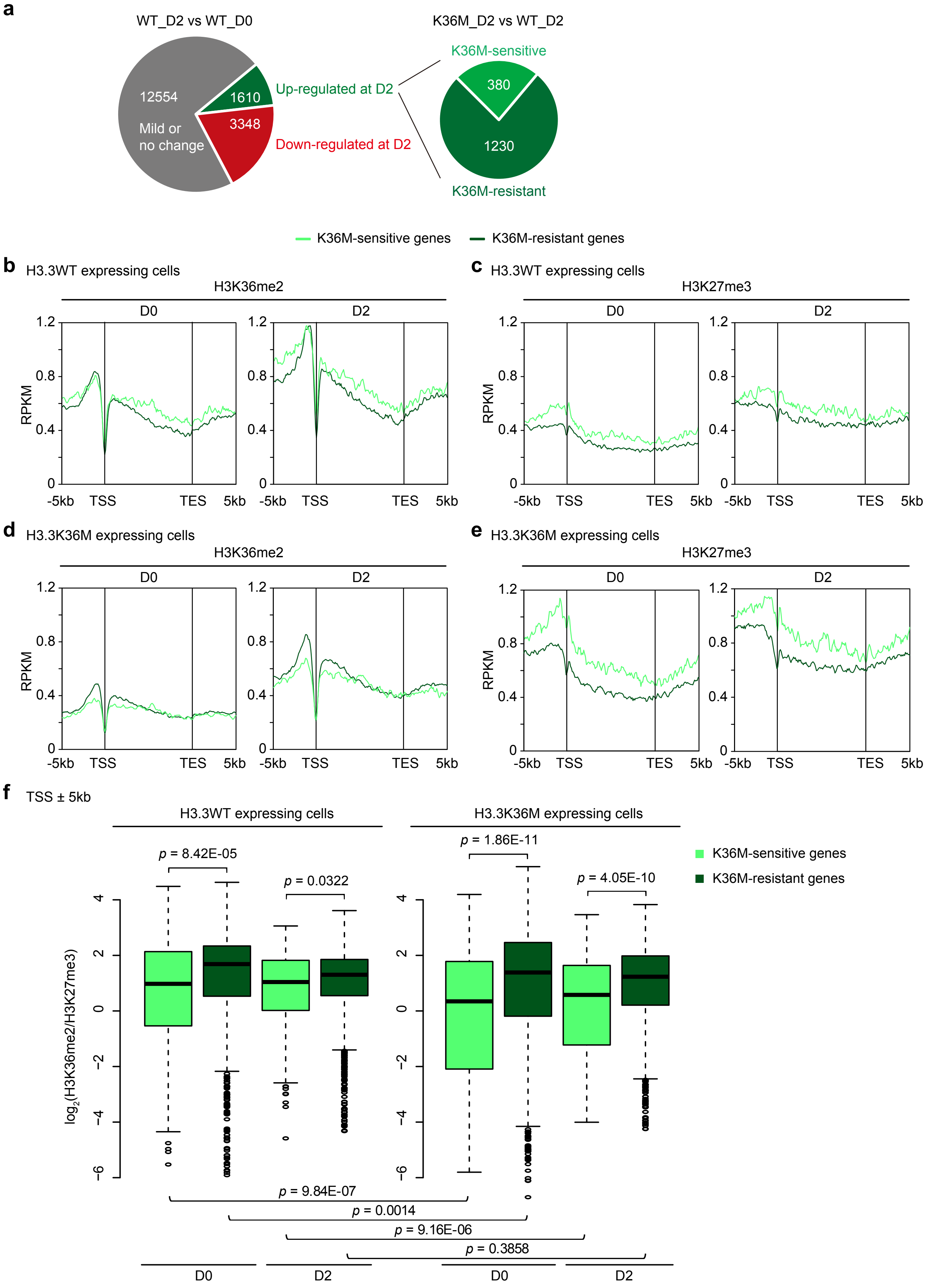
a
WT_D2 vs WT_D0
12554
Mild or no change
1610
Up-regulated at D2
3348
Down-regulated at D2
K36M_D2 vs WT_D2
K36M-sensitive
380
1230
K36M-resistant
K36M-sensitive genes
K36M-resistant genes
b H3.3WT expressing cells
H3K36me2
c H3.3WT expressing cells
H3K27me3
d H3.3K36M expressing cells
H3K36me2
e H3.3K36M expressing cells
H3K27me3
D0
D2
RPKM
-5kb TSS TES 5kb
f TSS ± 5kb
H3.3WT expressing cells
H3.3K36M expressing cells
p = 8.42E-05
p = 0.0322
p = 1.86E-11
p = 4.05E-10
K36M-sensitive genes
K36M-resistant genes
log2(H3K36me2/H3K27me3)
p = 9.84E-07
p = 0.0014
p = 9.16E-06
p = 0.3858
D0
D2
D0
D2

Supplementary Figure 3. H3K36me2 and H3K27me3 levels on K36M-sensitive or -resistant genes during adipogenesis. (**a**) Schematic of identification of K36M-sensitive and -resistant up-regulated genes at D2 of adipogenesis as shown in Figure 1d. (**b-e**) Average profiles of H3K36me2 and H3K27me3 on gene body of K36M-sensitive or -resistant genes in H3.3WT (**b-c**) or H3.3K36M (**d-e**) expressing cells at D0 (left panel) and D2 (right panel) of adipogenesis. (**f**) Boxplot shows the ratio of H3K36me2/H3K27me3 on TSS ± 5kb of K36M-sensitive or -resistant genes in H3.3WT (left panel) and H3.3K36M (right panel) expressing cells at D0 and D2 of adipogenesis. TSS, transcription start site; TES, transcription end site. Box plots show the median, the 25th and 75th percentiles, Tukey whiskers (median ± 1.5 times interquartile range), and outliers (◦). Statistical comparison between groups was performed using Student's *t* test.

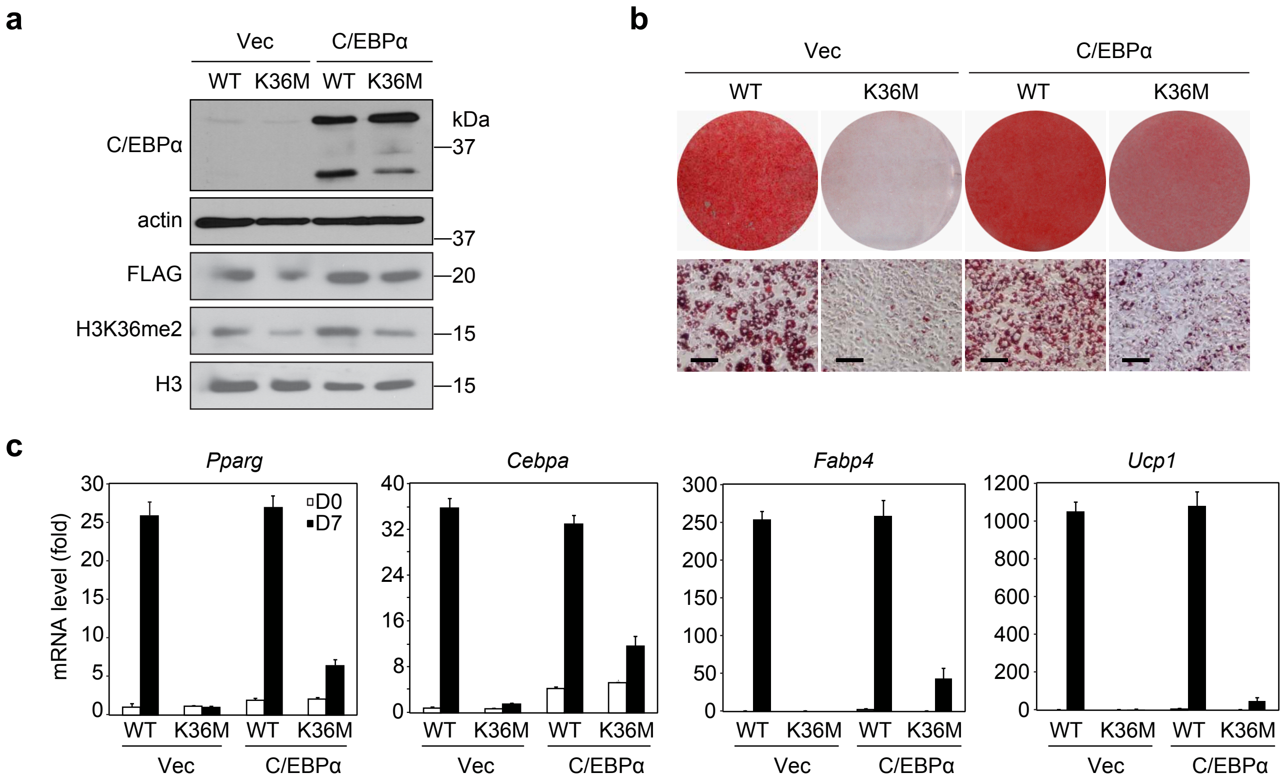

Supplementary Figure 4. H3.3K36M inhibits C/EBPα-stimulated adipogenesis. Immortalized brown preadipocytes were infected with retroviral vector pQCXIP expressing FLAG-tagged WT or K36M mutant histone H3.3. After puromycin selection, cells were infected with retroviral vector pWZLhygro expressing C/EBPα. After hygromycin selection, cells were induced to undergo adipogenesis. (**a**) Western blot analysis in preadipocytes using antibodies indicated on the left. (**b**) Oil Red O staining at D7 of adipogenesis. Scale bars = 30 μm. (**c**) qRT-PCR of *Pparg*, *Cebpa*, *Fabp4* and *Ucp1* expression at D0 and D7 of adipogenesis. qRT-PCR data are presented as means ± SEM. Three technical replicates from a single experiment were used.

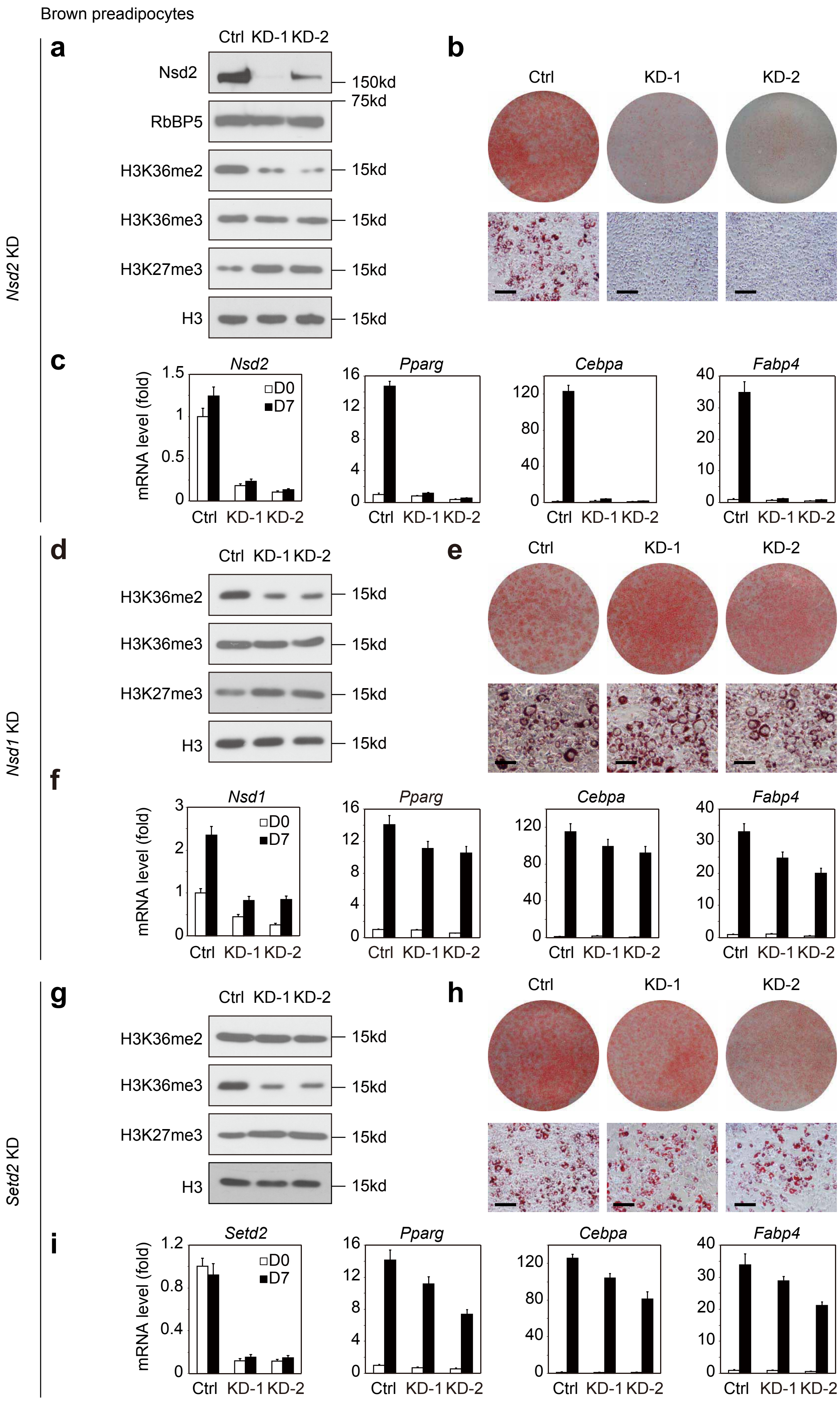

Brown preadipocytes
a
b
c
d
e
f
g
h
i
Nsd2 KD
Nsd1 KD
Setd2 KD
Ctrl
KD-1
KD-2
Nsd2
RbBP5
H3K36me2
H3K36me3
H3K27me3
H3
150kd
75kd
15kd
mRNA level (fold)
D0
D7
Nsd1
Setd2
Pparg
Cebpa
Fabp4

Supplementary Figure 5. H3K36 methyltransferase Nsd2 is essential for adipogenesis. Immortalized brown preadipocytes were infected with lentiviral vector expressing control (Ctrl) or knockdown (KD) shRNAs, followed by adipogenesis assay until day 7. (**a-c**) Nsd2 is required for adipogenesis. (**a**) Western blot of Nsd2 and histone methylations in preadipocytes. RbBP5 and histone H3 were used as loading controls. (**b**) Oil Red O staining at D7 of adipogenesis. (**a**) and (**b**) are also shown in Figure 3a-b. Scale bars = 30 μm. (**c**) qRT-PCR of *Nsd2*, *Pparg*, *Cebpa* and *Fabp4* expression at D0 and D7 of adipogenesis. (**d-f**) Nsd1 is largely dispensable for adipogenesis. (**d**) Western blot of histone methylations in preadipocytes. Histone H3 was used as loading controls. (**e**) Oil Red O staining at D7 of adipogenesis. Scale bars = 30 μm. (**f**) qRT-PCR of *Nsd1*, *Pparg*, *Cebpa* and *Fabp4* expression. (**g-i**) Setd2 is largely dispensable for adipogenesis. (**g**) Western blot of histone methylations in preadipocytes. (**h**) Oil Red O staining at D7 of adipogenesis. Scale bars = 30 μm. (**i**) qRT-PCR of *Setd2*, *Pparg*, *Cebpa* and *Fabp4* expression. All qRT-PCR data are presented as means ± SEM. Three technical replicates from a single experiment were used.

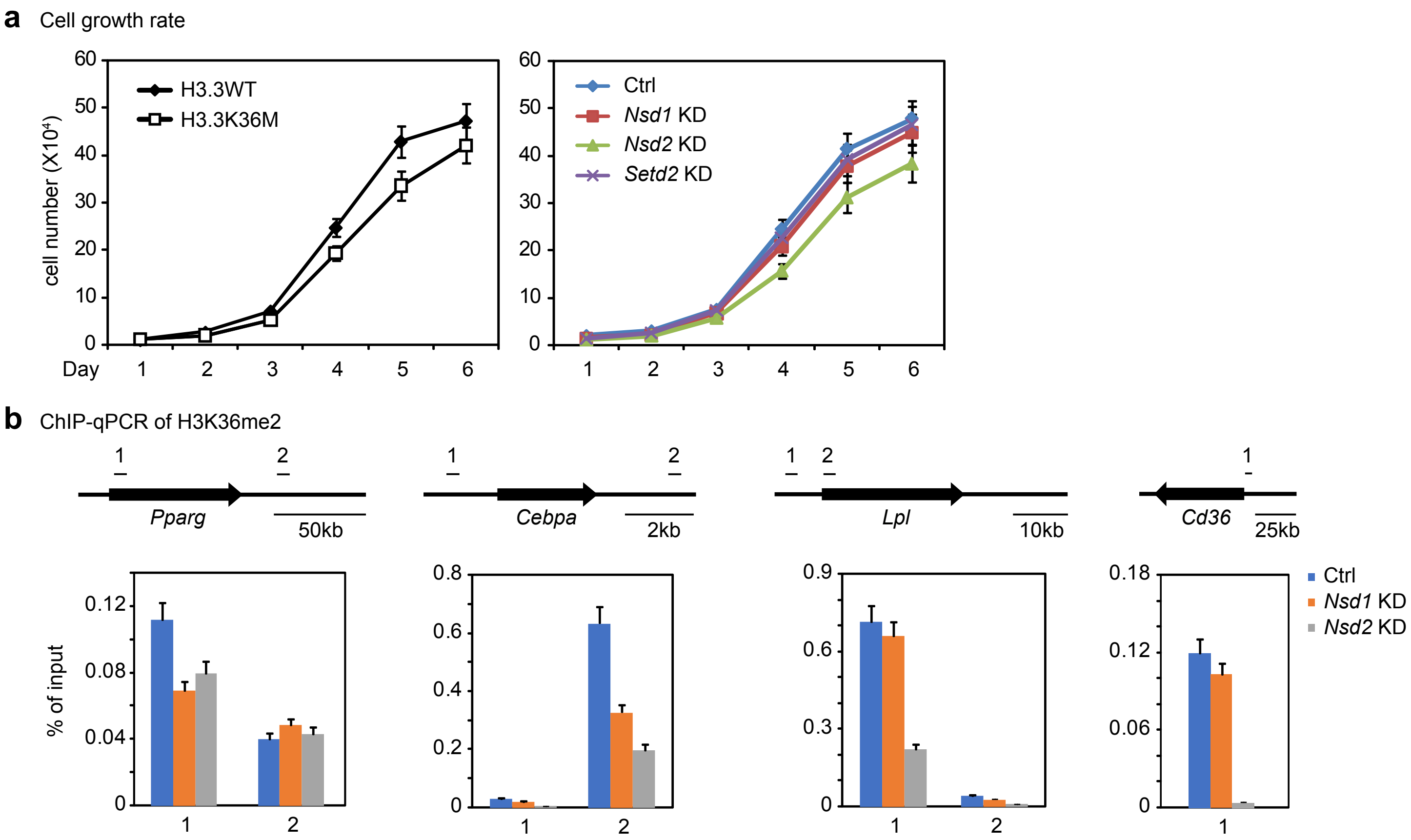

Supplementary Figure 6. Characterization of preadipocytes with ectopic expression of H3.3K36M or knockdown of H3K36 methyltransferases. (**a**) Cell growth rates. H3.3WT and H3.3K36M expressing cells shown in Figure 1 (left panel); Ctrl, *Nsd1* KD, *Nsd2* KD and *Setd2* KD cells shown in Figure 3 and Supplementary Figure 5 (right panel). (**b**) Levels of H3K36me2 around *Pparg*, *Cebpa*, *Lpl* and *Cd36* loci were measured by ChIP-qPCR in Ctrl, *Nsd1* KD and *Nsd2* KD preadipocytes. All values are presented as means ± SEM. Three technical replicates from a single experiment were used.

a

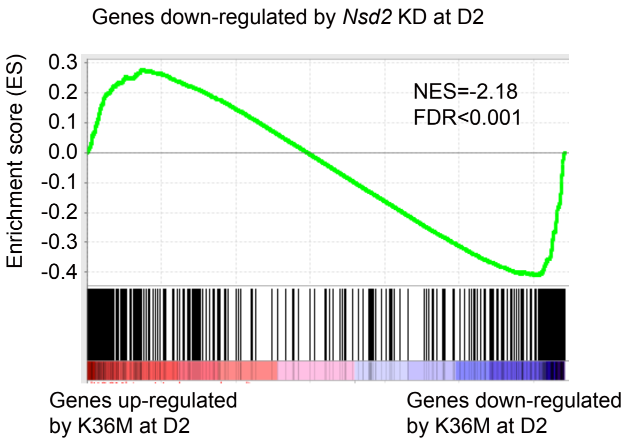

164 core enrichment genes

GO term	P value
brown fat cell differentiation	5.80E-07
fat cell differentiation	1.30E-06
positive regulation of response to external stimulus	7.10E-05
response to hormone stimulus	3.70E-04
glycerol ether catabolic process	4.80E-04
triglyceride catabolic process	4.80E-04

b

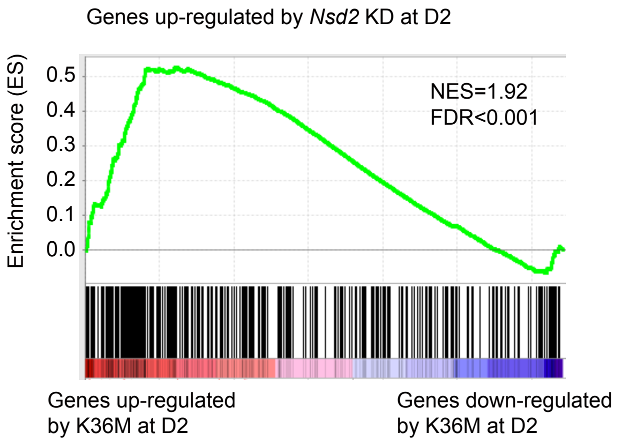

198 core enrichment genes

GO term	P value
cell adhesion	1.80E-06
biological adhesion	1.80E-06
cell motion	1.70E-05
tube development	3.90E-05
regulation of cell adhesion	3.00E-04
regulation of cell proliferation	5.10E-04

Supplementary Figure 7. Gene set enrichment analysis of genes regulated by *Nsd2* KD and ectopic H3.3K36M expression at D2 of adipogenesis. (**a**) Genes down-regulated by *Nsd2* KD are enriched in genes down-regulated by H3.3K36M at D2 of adipogenesis. GO analysis of the 164 core enriched genes is shown in the lower panel. (**b**) Genes up-regulated by *Nsd2* KD are enriched in genes up-regulated by H3K36M at D2 of adipogenesis. GO analysis of the 198 core enriched genes is shown in the lower panel. The enrichment plots are shown at the top. Black vertical lines indicate gene hits and the ranking metric scores based on the ratio of RNA-Seq results in WT and K36M cells at D2 of adipogenesis.

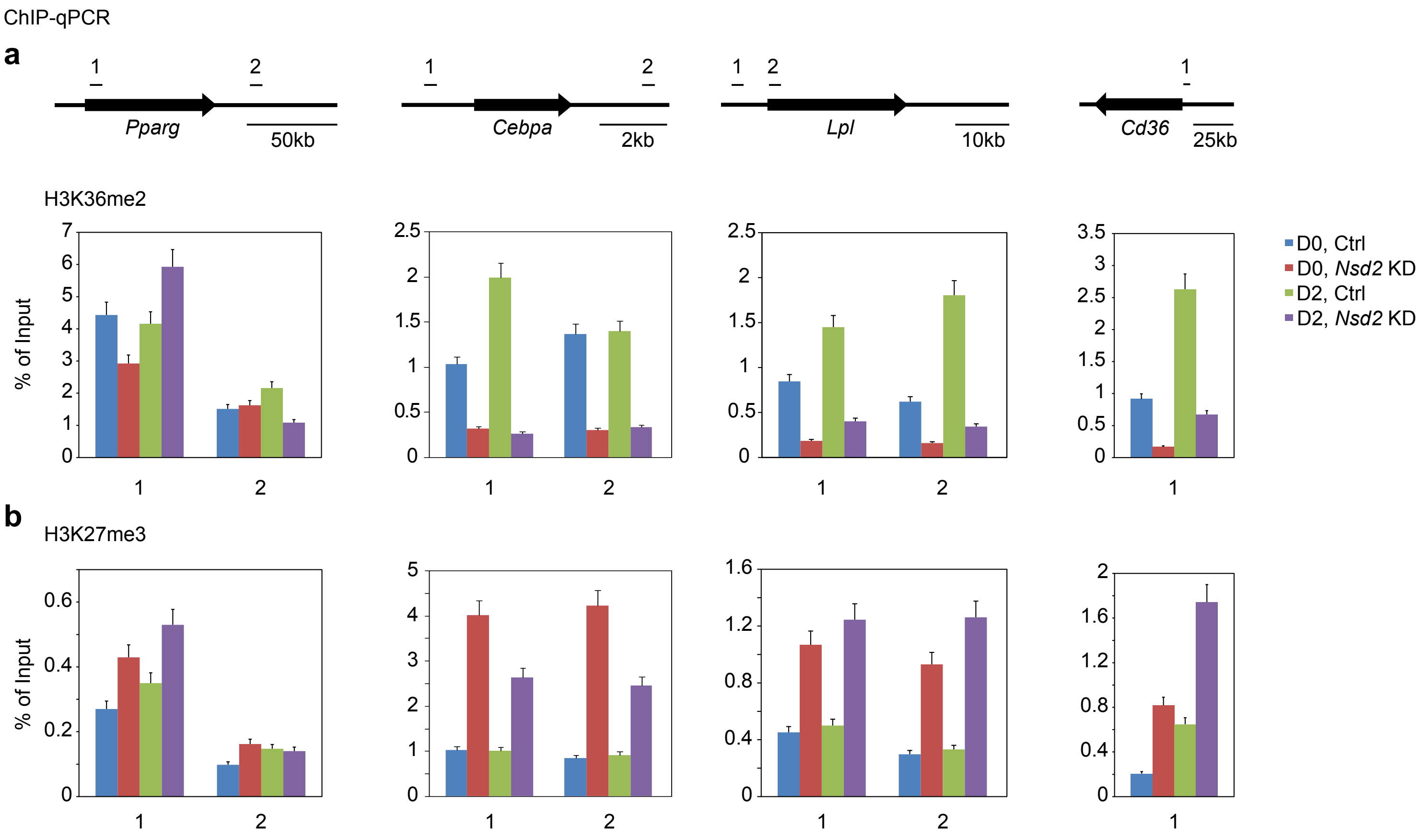

Supplementary Figure 8. Depletion of H3K36me2 by *Nsd2* KD increases H3K27me3 to prevent the induction of adipogenic genes. Immortalized brown preadipocytes were infected with lentiviral vector expressing Ctrl or *Nsd2* KD shRNAs as described in Figure 3, followed by adipogenesis assay. Levels of H3K36me2 (**a**) and H3K27me3 (**b**) around *Pparg*, *Cebpa*, *Lpl* and *Cd36* loci were measured by ChIP-qPCR at D0 and D2 of adipogenesis. Upper panels in (**a**) show the locations of the PCR primers. All values are presented as means ± SEM. Three technical replicates from a single experiment was used.

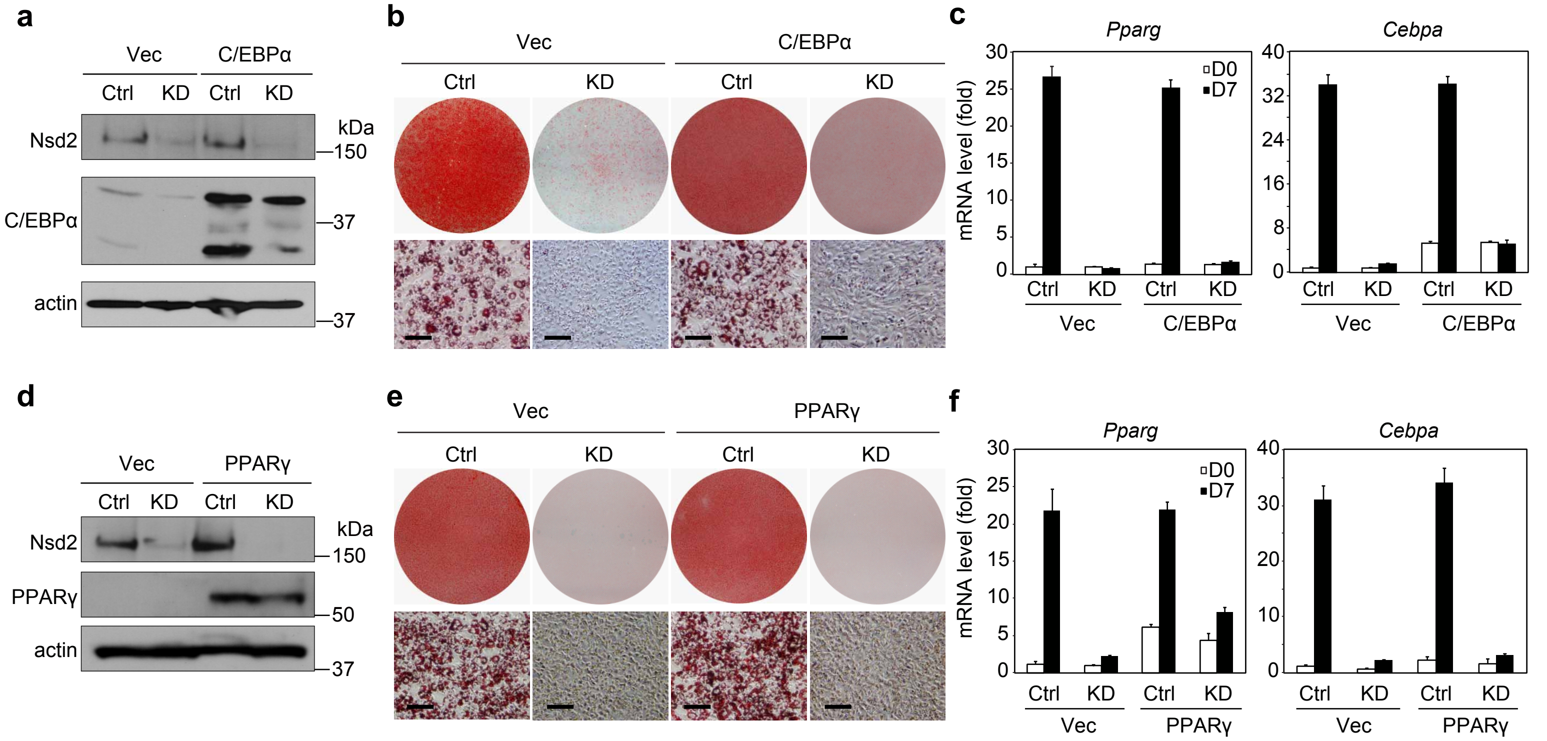

Supplementary Figure 9. *Nsd2* KD inhibits C/EBPα- or PPARγ-stimulated adipogenesis. Immortalized preadipocytes were infected with lentiviral vector expressing Ctrl or *Nsd2* KD shRNA. After puromycin selection, cells were infected with retroviral vector expressing C/EBPα (**a-c**) or PPARγ (**d-f**), followed by adipogenesis assay. (**a** and **d**) Western blot of Nsd2, C/EBPα and PPARγ in preadipocytes. (**b** and **e**) Oil Red O staining at D7 of adipogenesis. Scale bars = 30 μm. (**c** and **f**) qRT-PCR of *Pparg* and *Cebpa* expression at D0 and D7 of adipogenesis. qRT-PCR data are presented as means ± SEM. Three technical replicates from a single experiment were used.

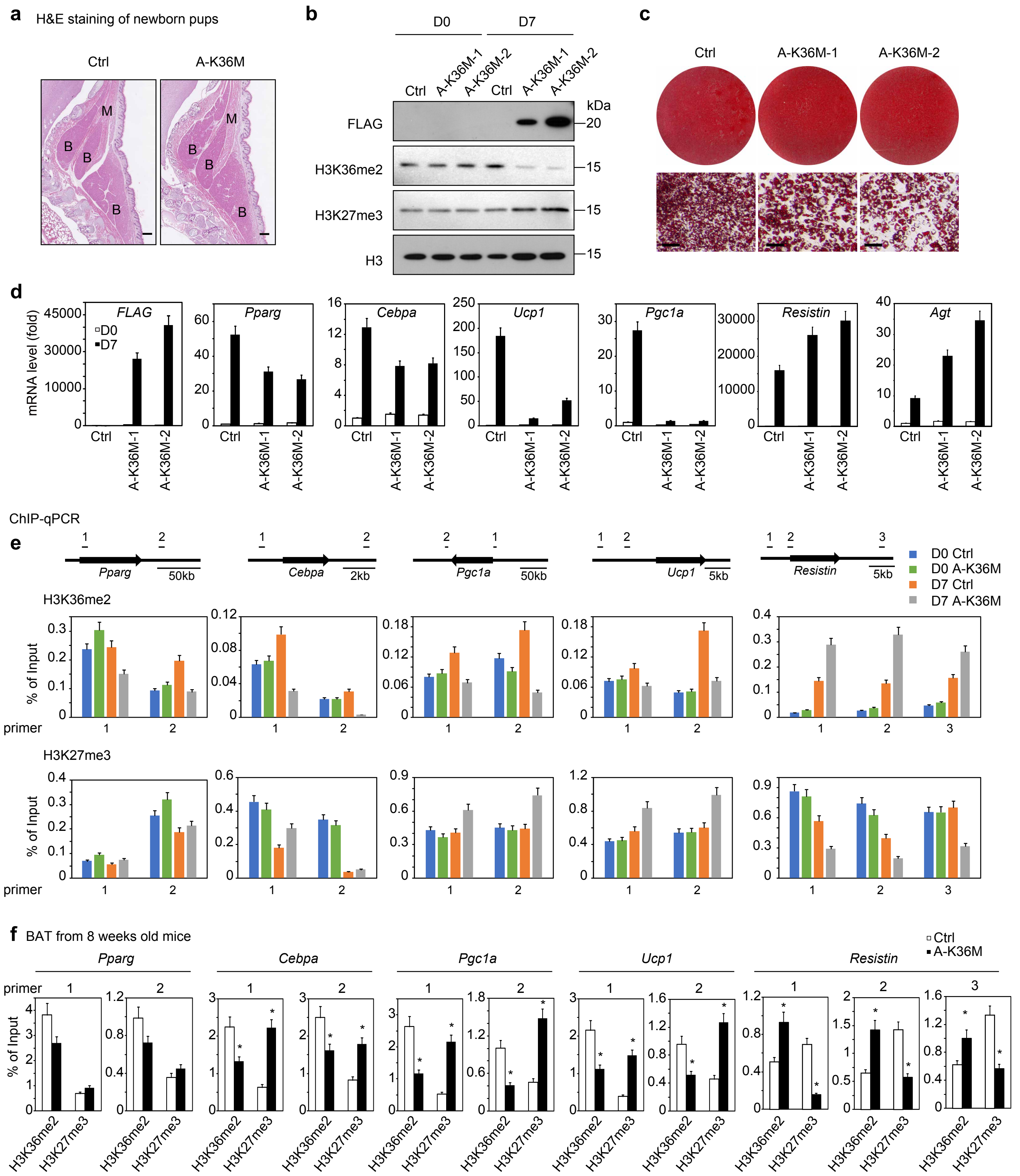

Supplementary Figure 10. *Fabp4* promoter-driven H3.3K36M expression in brown adipocytes reprograms gene expression. (**a**) Histological analysis of Ctrl or A-K36M newborn pups. Sagittal sections of cervical/thoracic area were stained with H&E. BAT (B); muscle (M). Scale bar = 300 µm. (**b-e**) Primary brown preadipocytes were isolated from the BAT of one Ctrl

and two A-K36M newborn pups, followed by adipogenesis assay. Cells were collected at indicated time points for Western blot, qRT-PCR and ChIP-qPCR. (**b**) Western blot analysis of histone extracts using FLAG and histone H3 antibodies. (**c**) Oil Red O staining at D7 of adipogenesis. Scale bars = 30 µm. (**d**) qRT-PCR of gene expression at D0 and D7 of adipogenesis. (**e**) Levels of H3K36me2 and H3K27me3 around *Pparg*, *Cebpa*, *Pgc1a*, *Ucp1* and *Resistin* loci in primary brown preadipocytes at D0 and D7 of adipogenesis were measured by ChIP-qPCR. (**f**) Levels of H3K36me2 and H3K27me3 around *Pparg*, *Cebpa*, *Pgc1a*, *Ucp1* and *Resistin* loci in the BAT of 8 weeks old Ctrl and A-K36M mice were measured by ChIP-qPCR. Upper panels in (**e**) show the locations of the qPCR primers used in (**e**) and (**f**). All values are presented as means ± SEM. Three technical replicates from a single experiment were used.

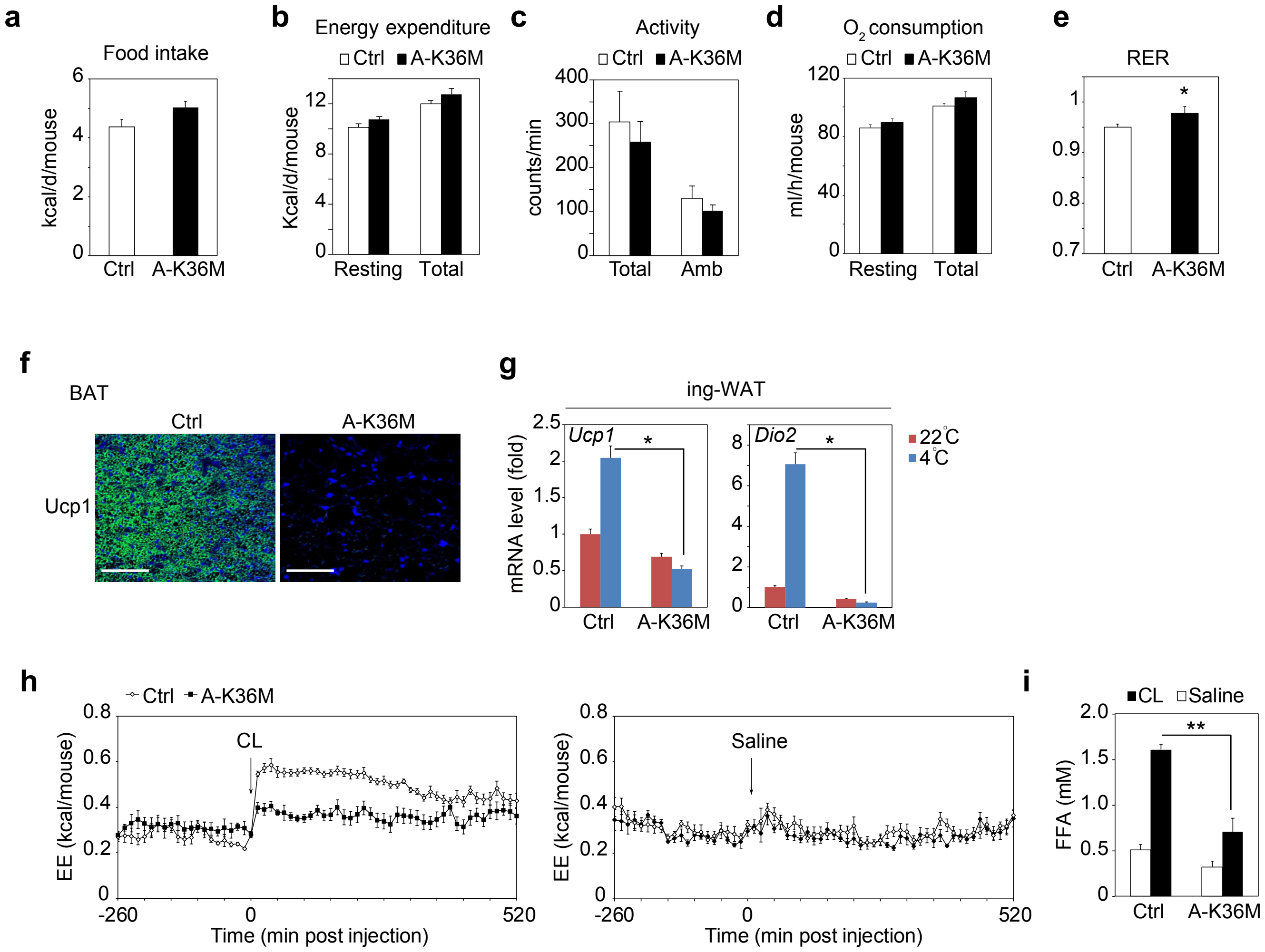

Supplementary Figure 11. Mice with adipose-selective expression of H3.3K36M show severe defects in thermogenesis and lipolysis. 8~10 weeks old male Ctrl and A-K36M mice (*n* = 6 per group) were fed with regular diet. (**a-e**) Food intake (**a**), resting and total energy expenditure (**b**), average total and ambulatory (Amb) activity (**c**), resting and total O_2 consumption (**d**), and respiratory exchange ratios (RER) over three consecutive days (**e**) were measured by CLAMS. (**f**) BAT sections were immunostained for Ucp1. Scale bar = 100 µm. (**g**) Mice were housed at room temperature (22°C) or in a cold room (4°C) for 2h as described in Figure 5. *Ucp1* and *Dio2* expression in ing-WAT was determined by qRT-PCR. (**h**) Total energy expenditure (EE) of mice injected with CL316,243 (CL) (left) or saline (right). (**i**) Serum levels of free fatty acid (FFA) 20 min after saline or CL administration. All values in Supplementary Figure 11 are presented as means ± SEM. Statistical comparison between groups was performed using Student's *t* test. * p<0.05, ** p<0.01.

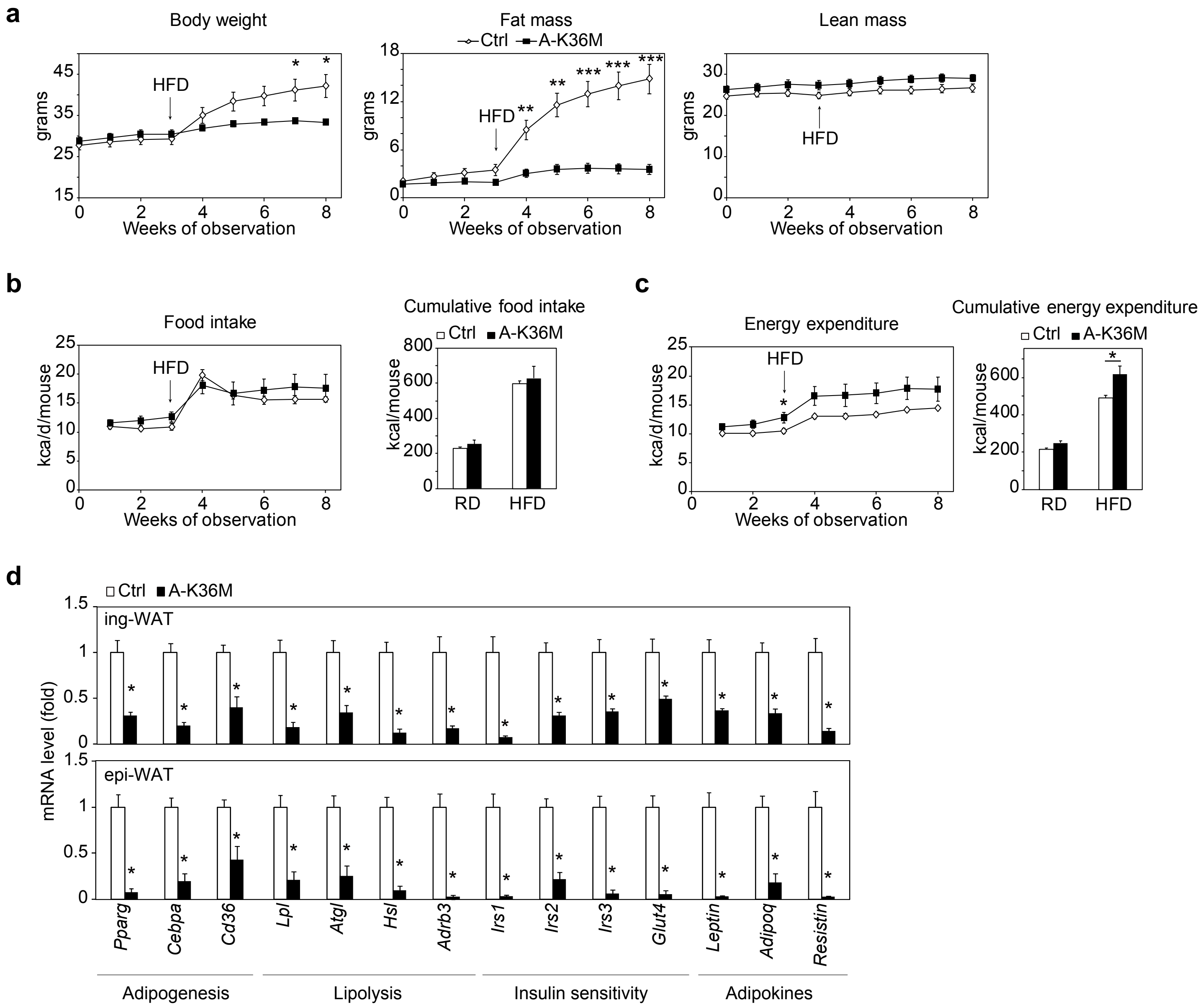

Supplementary Figure 12. Further characterization of mice with adipose-selective expression of H3.3K36M after high fat diet. (**a-c**) Continuous analysis of body composition (**a**), food intake (**b**) and energy expenditure (**c**). 8 weeks old Ctrl and A-K36M mice (*n* = 6 per group) were kept on regular diet (RD) for 3 weeks. After that, mice were fed with HFD for 5 weeks. (**d**) qRT-PCR analysis of adipogenesis, lipolysis, insulin sensitivity and adipokine genes in ing-WAT and epi-WAT of Ctrl and A-K36M mice (*n* = 6 per group) after HFD. All values are presented as means ± SEM. Statistical comparison between groups was performed using Student's *t* test. * $p<0.05$, ** $p<0.01$, *** $p<0.005$.

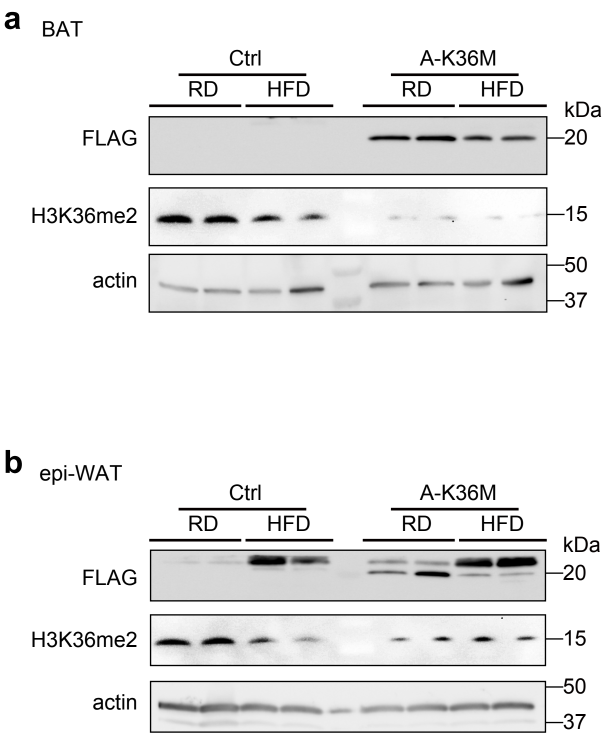

Supplementary Figure 13. HFD reduces H3K36me2 levels in mouse adipose tissues. Western blot of H3K36me2 in the BAT (**a**) or epi-WAT (**b**) of Ctrl and A-K36M mice under RD or HFD.

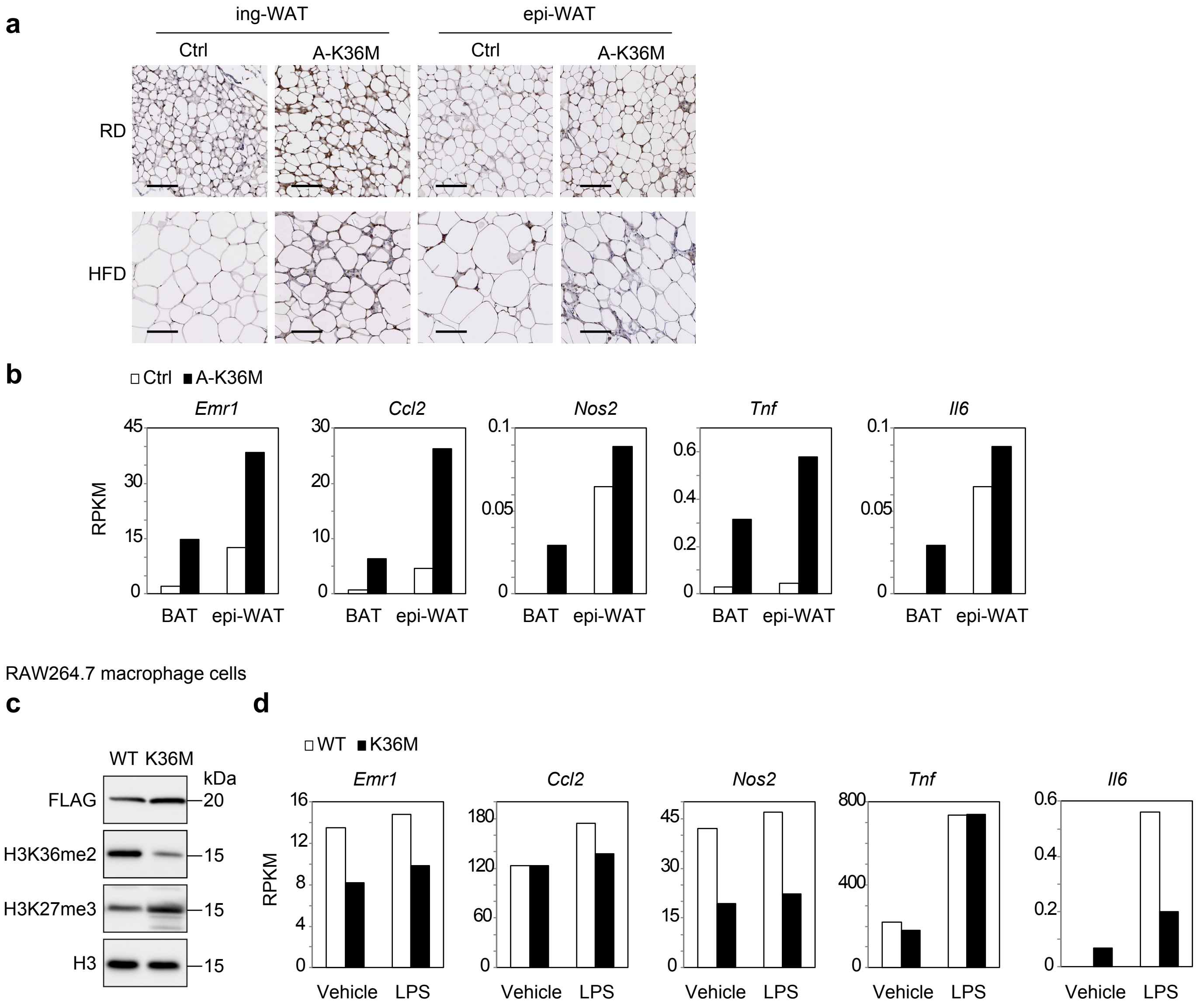

Supplementary Figure 14. Inflammatory response in A-K36M mice. (**a-b**) Inflammation in WATs of Ctrl and A-K36M mice. (**a**) Histological analysis of WATs stained with antibodies against macrophage marker F4/80. Scale bar = 100μm. (**b**) RPKM values of representative inflammatory response genes in BAT or epi-WAT of Ctrl and A-K36M mice. (**c-d**) RAW264.7 macrophage cells were infected with retroviral vector pQCXIP expressing WT or K36M of histone H3.3. Cells were stimulated with LPS (100 ng mL^{-1}) for 1h, followed by RNA-Seq analyses. (**c**) Western blot of FLAG-tagged histone H3.3 and H3K36me2. (**d**) RPKM values of representative inflammatory response genes in RAW264.7 cells.

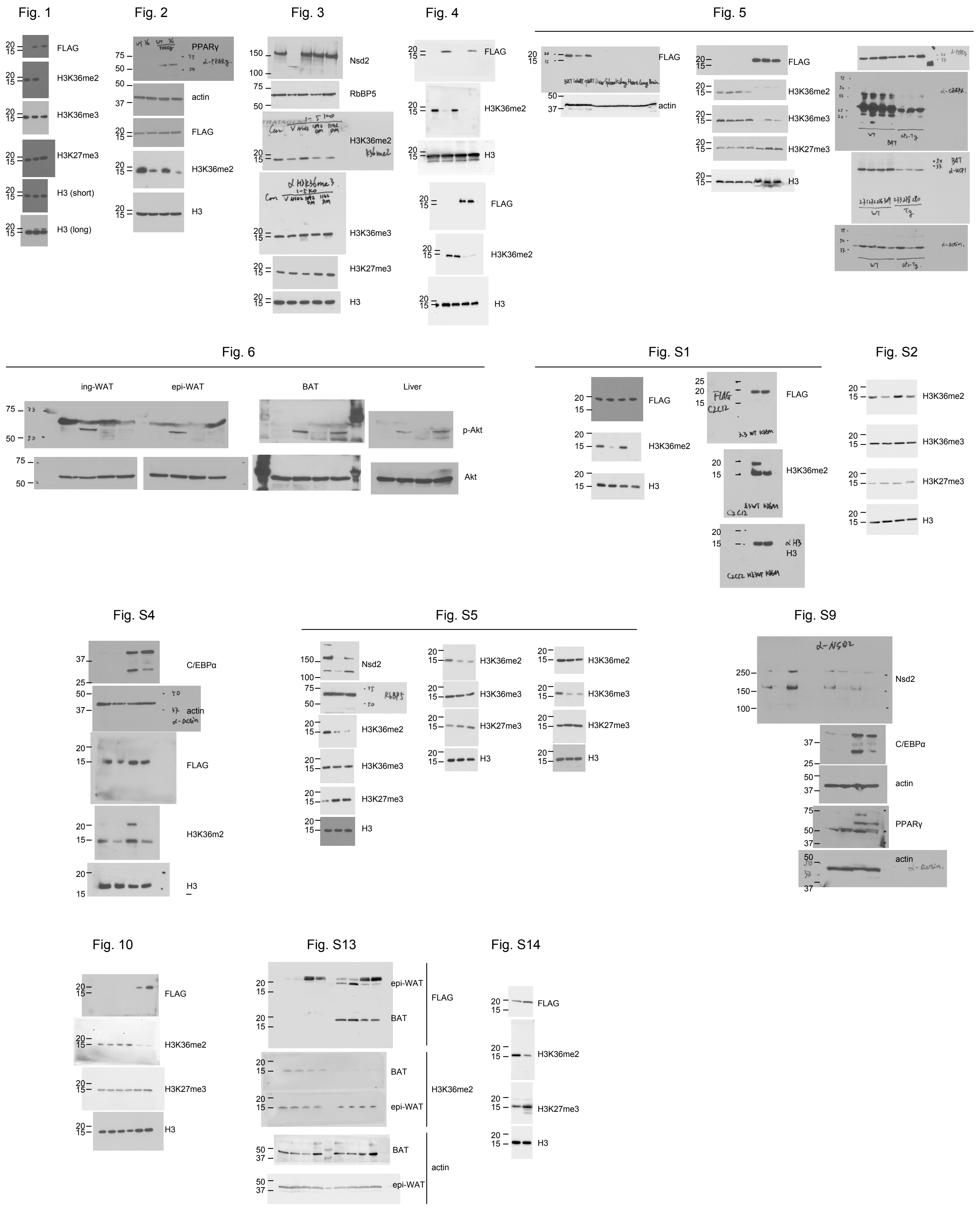

Supplementary Figure 15. Uncropped blots

qRT-PCR	Forward	Reverse
Pparg	TCGCTGATGCACTGCCTATG	GAGAGGTCCACAGAGCTGATT
Cebpa	CAAGAACAGCAACGAGTACCG	GTCACTGGTCAACTCCAGCAC
Cd36	GGTCCTTACACATACAGAGTTCGTTAT	CATTGGGCTGTACAAAAGACACA
Fabp4	GGGGCCAGGCTTCTATTCC	GGAGCTGGGTTAGGTATGGG
Lpl	GGGAGTTTGGCTCCAGAGTTT	TGTGTCTTCAGGGGTCCTTAG
Hsl	CCAGCCTGAGGGCTTACTG	CTCCATTGACTGTGACATCTCG
Atgl	CAACGCCACTCACATCTACGG	GGACACCTCAATAATGTTGGCAC
Adrb3	GGCCCTCTCTAGTTCCCAG	TAGCCATCAAACCTGTTGAGC
Leptin	GAGACCCCTGTGTCGGTTC	CTGCGTGTGTGAAATGTCATTG
Adipoq	GTCTGTACGATTGTCAGTGGATCTG	AAGAGGAACAGGAGAGCTTGCA
Resistin	AAGAACCTTTCATTTCCCCTCCT	GTCCAGCAATTTAAGCCAATGTT
Irs1	CGATGGCTTCTCAGACGTG	CAGCCCGCTTGTTGATGTTG
Irs2	CTGCGTCCTCTCCCAAAGTG	GGGGTCATGGGCATGTAGC
Irs3	TCGGCTCACCGTTTCCTTG	TCGCTCTCGTAGCACTCCA
Glut4	GTGACTGGAACACTGGTCCTA	CCAGCCACGTTGCATTGTAG
Agt	TCTCCTTTACCACAACAAGAGCA	CTTCTCATTCACAGGGGAGGT
Ucp1	GGCCTCTACGACTCAGTCCA	TAAGCCGGCTGAGATCTTGT
Dio2	CAGTGTGGTGCACGTCTCCAATC	TGAACCAAAGTTGACCACCAG
Pgc1a	CCCTGCCATTGTTAAGACC	TGCTGCTGTTCCTGTTTTC
Prdm16	CAGCACGGTGAAGCCATTC	GCGTGCATCCGCTTGTG
Nsd1	ATTTGGGCAAAATTCAAGAGACG	GCCTCCTATTGGCAACTTTCATT
Setd2	CACCAGGTTACCCCATGCAAG	TCTGTAGAATGTCCTACCAAGGG
H3FLAG	AACCTGTGTGCCATCCACG	CGACTTGTCATCGTCGTCCTT
18S	ATGCCCTGCCCTTTGTACACA	CGATCCGAGGGCCTCACTA

ChIP-qPCR	Forward	Reverse
Pparg1	AGGTGACTGGGTCTCCGCT	TCGAGACAGGGTTAGGCTGTC
Pparg2	GCTGTGGTGAAACGACAGTTATTAG	CTTGGGAGCTACAGCCTTGTG
Cebpa1	CACGGACAAATGGAGTTCGA	CCACGTCTCTCGTTTTTGCA
Cebpa2	ATAGACATCAGCGCCTACATCGA	GTCGGCTGTGCTGGAAGAG
Lpl1	GCGCTCCACCTTGTTAGTCT	GGGCTTAGGAGTTTGCTGGT
Lpl2	GCACTGCTCAAACAACCAGG	GCAGGCCTTCGAGGTGTTAT
Cd36	AGCACTTGAGAACGCCTGAA	GGCTCAGGAATGGGAGTGTC
Pgc1a1	TGCCTCAGTGAAGTAACGCT	TCCGGTTTAGAGTTGGTGGC
Pgc1a2	TGCCTCAGTGAAGTAACGCT	TCCGGTTTAGAGTTGGTGGC
Ucp11	GCTGTCTACTAGGCTCTCCACTCA	TCGGCTGCCTCTGCAAAG
Ucp12	TTGAGAATTTCCTTCCCTAATGCT	GCAGAGGGCTCACAAACAGAA
Resistin1	CCGGGTGAGACTAGGGGTAT	CATCCCCCACCTTTGTGCTA
Resistin2	AGTCAGAGTGCTGATGTGGC	CGTGGGTACTGCAAGAGACA
Resistin3	ACAGCTCTCCGTCTAACCCT	TTTCTTGGGACCAATGGGGG

Supplementary Table 1. Primers used for quantitative PCR
